# Assessment of Quality of Life in Patients of Mastectomy With Chemotherapy

**DOI:** 10.7759/cureus.27703

**Published:** 2022-08-05

**Authors:** Vaishnavi P Deshpande, Raju K Shinde, Deepali Deo, Prashant Hippargekar, Shreya V Venurkar

**Affiliations:** 1 Department of General Surgery, Jawaharlal Nehru Medical College, Datta Meghe Institute of Medical Sciences, Wardha, IND; 2 Department of Community Medicine, Swami Ramanand Teerth Rural Government Medical College, Ambajogai, IND; 3 Department of Otorhinolaryngology, Swami Ramanand Teerth Rural Government Medical College, Ambajogai, IND; 4 Department of Psychiatry and Behavioral Sciences, Jawaharlal Nehru Medical College, Datta Meghe Institute of Medical Sciences, Wardha, IND

**Keywords:** economic status, psychological, spiritual, physical, quality of life, demography, fnac, breast reconstruction, breast cancer

## Abstract

Background: Breast cancer is one of the most common cancers in India as well as the world. In India, 48% of patients with breast cancer are below 50 years of age, indicating a huge age shift in the last 25 years. Breast cancer in an early age group increased the five-year survival rate and increased life expectancy has created a large group of breast cancer survivors who battle scars of disease as well as treatment. Standardized multimodal treatment is either not affordable or not available, so the breast conservation surgery rate is very low. Mastectomy is still the most common modality of treatment, particularly in rural areas. In addition to psychological, social, economic, and family barriers to obtaining the diagnosis and treatment needed, economic barriers like the cost of travel and lost wages are important factors influencing the choice of treatment. Mastectomy represents a deep burden for women with breast cancer. Very little is known about the psychological consequences over time and the quality of life (QOL) of women so treated, with or without breast reconstruction. Conflicting literature is available regarding QOL after mastectomy. The survival rates of breast cancer are increasing. They are reported in the range of 80-90% in western countries while in the range of 60% in the Indian scenario. With high survival rates in cancer, the focus needs to shift from mortality indicators to QOL indicators. The QOL that these survivors experience is a comparatively newer domain of study. Though there are many instruments for assessment of breast QOL of breast cancer patients with numerous studies in western literature, QOL studies in Indian rural population are far less, and urban studies cannot be extrapolated because the method of treatment differs, with breast conservation being more common in urban population. Hence, the present study is undertaken to assess the QOL in patients who have undergone mastectomy and ongoing chemotherapy or completed chemotherapy recently using a relatively newer instrument, i.e., the Quality of Life Instrument - Breast Cancer Patient version.

Methodology: The present study was a cross-sectional study conducted at a rural tertiary healthcare center on mastectomy patients attending the outpatient department and admitted to the hospital. All the female patients of carcinoma breast treated with a mastectomy who were receiving the adjuvant or neoadjuvant chemotherapy or were within one year of completion of chemotherapy irrespective of age at diagnosis were included in this study. The assessment was performed by interview method using a questionnaire.

Results: In this study, 44.90% of the patients were <50 years old and 55.10% were more than 50 years old. Among them, 28.57% were illiterate while only 20.41% had graduate education. The majority (61.22%) were from the low socioeconomic class. Majority of women presented in the late stages of the disease, with 61.22% presenting in the third stage and only three (6.12%) presenting in the first stage of the disease. The overall global QOL score was 49 ± 2.6 and fear was assessed. Patients scored better in the physical, psychological, social, and spiritual domains, with an average score of more than 50. The worst scores were observed among distress of illness or treatment.

Conclusions: The present study shows that the average QOL scores in rural Indian women after mastectomy are moderate. Global scores and other indicators show moderate QOL.

## Introduction

Breast cancer is the most common cancer in urban Indian females and the second most common in rural Indian women because of rising incidence and awareness [1]. In India, we are also witnessing more and more numbers of patients being diagnosed with breast cancer to be in the younger age groups (in their 30s and 40s) [2]. At present, 48% of patients with breast cancer are below 50 years of age, indicating a huge age shift in the last 25 years when only 31% were below 50 years of age. The absolute incidence rate of breast cancer is 25.8 per lakh in India and only 24% of them present in an early stage with the rest presenting with advanced disease [3,4]. Breast cancer in an early age group increased the five-year survival rate and increased life expectancy has created a large group of breast cancer survivors who battle scars of disease as well as treatment. Detection of second breast cancers in the asymptomatic phase leads to detection of early-stage cancer and improves relative survival by between 27% and 47% [5].

There is a huge gap in presentation pattern, survival rate, and availability of modern treatment in rural and urban areas. Rural women present late in course of the disease. Standardized multimodal treatment is either not affordable or not available, so the breast conservation surgery rate is very low [6]. Mastectomy is still the most common modality of treatment, particularly in rural areas. In addition to psychological, social, economic, and family barriers to obtaining the diagnosis and treatment needed, economic barriers like the cost of travel and lost wages are important factors influencing the choice of treatment [6].

Other factors leading to increased rates of mastectomy in rural areas are large tumor size at presentation, lack of services like sentinel node biopsy, frozen section, patients' concern regarding recurrence, avoiding possible further treatment, and inability to spend longer time for treatment [7]. The decision of mastectomy though appears straightforward, it has a huge psychological impact on quality of life (QOL). Mastectomy represents a deep burden for women with breast cancer. Very little is known about the psychological consequences over time and the QOL of women so treated, with or without breast reconstruction (BR) [8]. Conflicting literature is available regarding QOL after mastectomy. The breast is considered the pride of motherhood, femininity, a symbol of sexuality, and attractiveness. Mastectomy leads to loss of breast and physical disfigurement. The adjuvant treatment adds to hair loss, initiation of menopause, and vaginal dryness. These changes lead to isolation from social and personal relations due to feelings of incompleteness as a female [9]. Cancer-related fatigue (CRF) has been documented as one of the most distressing symptoms reported by breast cancer survivors [10].

It is reported that 52% of females agreed there is no change in body image and their QOL in all the four domains of life, i.e., physical, social, psychological, and environmental [11]. The survival rates of breast cancer are increasing. Five years after diagnosis, young breast cancer survivors who remained cancer-free enjoyed good health and improved QOL. Nonetheless, physical, social, and psychological concerns must be addressed so that young breast cancer survivors will continue to be resilient as they age [12]. They are reported in a range of 80-90% in western countries while in the range of 60% in the Indian scenario [2,3]. In countries like India where survival is the problem, main efforts are channelized to treatment, which needs to be shifted to the QOL. There are many factors like age at presentation, pre-radiation chemotherapy, treatment duration, body mass index (BMI), type of surgery, number of nodes removed and involved, and prior radiotherapy that affect the QOL of survivors of breast cancer [13,14]. Breast cancer patients with stage III disease showed significantly poorer functioning in all areas except family than did other breast cancer patients; however, when compared with the breast cancer screening group, they showed higher QOL scores in several domains [15]. Long-term survivors of breast cancer who had received diagnoses at an older age (>65 years) showed significantly worse QOL outcomes in the physical domain (p < 0.05), while those who had received diagnoses at a younger age (27-44 years) showed worse QOL outcomes in the social domain than other age groups [16].

With high survival rates in cancer, the focus needs to shift from mortality indicators to QOL indicators. The QOL that these survivors experience is a comparatively newer domain of study. The focus on the QOL is just emerging. There are many instruments for assessing breast QOL of breast cancer patients with numerous studies in the western literature. However, QOL studies in the Indian rural population are far less, and urban studies cannot be extrapolated because the method of treatment differs, with breast conservation being more common in the urban population.

Hence, the present study is undertaken to assess the QOL in patients that have undergone mastectomy and ongoing chemotherapy or completed chemotherapy recently using a relatively newer instrument, i.e., the QOL breast cancer version [17].

## Materials and methods

Materials

The current study was conducted at a rural tertiary healthcare center. It is a cross-sectional type of study. The study population taken into consideration were all the mastectomy patients attending the outpatient department (OPD) and admitted to the hospital. Inclusion criteria were all the female patients with carcinoma breast treated with a mastectomy who were receiving the adjuvant or neoadjuvant chemotherapy or were within one year of completion of chemotherapy irrespective of age at diagnosis. Similarly, exclusion criteria included patients with breast conservation surgery, male patients with carcinoma breast, mastectomy performed for other reasons than malignancy, patients with mastectomy without chemotherapy, and patients with metastatic disease who underwent mastectomy and chemotherapy.

Thus, the final sample size on which the study was conducted was 49 patients. This study was started after the approval of the protocol by the Institutional Ethics Committee. The study was conducted after obtaining the written informed consent of the patients.

Method

In the present study, only female patients of all age groups with operable breast malignancy confirmed on fine needle aspiration cytology (FNAC) or true cut histopathology were included. Diagnosed patients who underwent mastectomy with chemotherapy were included. All the eligible patients with carcinoma breast giving consent for participation were registered for assessment. The assessment was performed by interview method using a questionnaire by the principal investigator. For illiterate people, the interviewer read and explained the questionnaire to the patient and their response was noted by the facilitator. The data were collected using a pretested questionnaire in patients' language, internal validity was tested, and Cronbach's alpha was not calculated. The questionnaire included sociodemographic characteristics of participants such as age, education status, occupation, socioeconomic status, religion, marital status, and perceptions of patients regarding received treatment and views regarding breast conservation surgery, etc.

Socioeconomic status was assessed using the BG Prasad Classification. The BG Prasad Classification is used for computing the socioeconomic status of both urban and rural populations. It classifies the population into five classes; class I with the highest per capita income and class V with the lowest per capita income. Further grouping of classes I and II together as high socioeconomic status and classes IV and V together as low socioeconomic status, with class II as middle status has been used in the present study. The BG Prasad Classification needs to be modified as per the All India Consumer Price Index Modification for May 2021 and has been used as a reference in the present study [18].

QOL assessment was performed by using the Quality of Life Instrument - Breast Cancer Patient (QOL BC) version by the City of Hope National Medical Center and Beckman Research Institute [19]. A questionnaire about treatment perception of local centers and related to the option of breast conservation surgery at specialized centers was assessed. The instrument includes 46 items representing the four domains of QOL including physical, psychological, social, and spiritual well-being [17]. The questionnaire used for the study has been included in the Appendix. The analysis will be done in six different aspects, namely, physical problem, psychological well-being, distress, fear, social well-being, and spiritual well-being, including items 1-8, 9-18, 19-25, 26-30, 31-39, and 40-46, respectively. The questionnaire was administered by interview method and the patient was asked to denote the number to the degree with which she agrees or disagrees with the statement and the response was noted by the facilitator. Zero was the worst occurrence and 10 was the best outcome. Several items had reverse anchors and therefore these items’ reversal of score by subtraction from 10 was included in the coding. The items to be reversed are 1-7, 9, 10, 17-29, 31, 33-39, and 43. QOL scores for each aspect were calculated separately. Then the total score for each aspect was calculated by the addition of all scores in the aspects. The total scores were normalized by the following standard normalization formula for standardization to a scale of 0-100: (sum all items - m x j) x (100/(m x (k - j)); where m = number of items, j = minimum value an item can take, and k = maximum value an item can take [20].

Standardized scores for each aspect were calculated. An attempt was made to identify facts that are frequently affecting QOL perceptions in patients. QOL scores were assessed further according to sociodemographic variables. Statistical procedures were carried out in two steps. The first was data compilation and presentation, and the second was statistical analysis. In data compilation and presentation, the collected data were compiled systematically. A master table was prepared and the dataset was subdivided and distributed meaningfully and presented as individual tables on a Microsoft Excel worksheet (Microsoft Corporation, Redmond, WA). Later, statistical analysis was done with IBM Statistical Package for the Social Sciences (version 21.0; IBM Corp., Armonk, NY). Mean and standard deviation (SD) were calculated and the analysis of variance (ANOVA) test was applied to assess the relation of age, religion, marital status, stage of disease, and socioeconomic status to QOL. For all tests, p < 0.05 was considered for statistical significance.

## Results

The study was conducted in a rural teaching hospital in central India. The patients' demographic parameters and disease presentation were recorded. From the data collected, 44.90% of the patients were <50 years old and 55.10% were more than 50 years old. Nearly 65.31% of women were married and living with family, 22.45% were widowed, and 12.24% were divorcees. Among these women, 28.57% were illiterate while only 20.41% had graduate education. The majority (61.22%) were from the low socioeconomic class. Majority of women presented in the late stages of the disease with 61.22% presenting in the third stage and only three (6.12%) presenting in the first stage of the disease (Table 1).

**Table 1:** Distribution of patients according to the demographic profile

Sociodemographic characteristics	Total patients	
Age	Number	Percentage
<50 years	22	44.90
>50 years	27	55.10
Religion		
Hindu	30	61.22
Buddhist	11	22.45
Muslim	05	10.20
Others	03	6.12
Education		
Illiterate	14	28.57
Primary	11	22.45
Secondary	14	28.57
Graduate	10	20.41
Socio economic classification		
High	4	8.16
Middle	15	30.61
Lower	30	61.22
Marital status		
Married	32	65.31
Divorcee	06	12.24
Widowed	11	22.45
Stage of disease		
First stage	03	6.12
Second stage	16	32.65
Third stage	30	61.22

The overall global QOL score was 49 ± 2.6. The worst scores were when distress and fear were assessed. The patient scored better in the physical, psychological, social, and spiritual domains with average scores of more than 50. There was no statistically significant difference found according to age, religion, education, marital status, stage of disease, or socioeconomic status (Table 2).

**Table 2:** Quality of life scores of patients in different domains

Sr. No.	Indicators	Mean	Standard deviation
1	Physical well-being	51.43	5.39
2	Psychological well-being	55.31	6.58
3	Distress of treatment or illness	36.91	6.8
4	Fear	47.82	3.65
5	Social well-being	51.04	6.9
6	Spiritual well-being	50.64	6.48
7	Global quality of life score	49.47	2.6

In Table 3, there were eight questions about the physical problems in the QOL instrument. Vaginal dryness was the main problem with mean scores of 1.20 ± 0.87. This was followed by appetite change (5.18 ± 2.68). The patients had fewer problems with menstrual changes, weight gain, etc. Patients rated overall physical health at 6.51 on a scale of 10.

**Table 3:** Distribution of quality of life scores of patients according to age

Sr. No.	Age	Age < 50 years (N = 22)		Age > 50 years (N = 27)		ANOVA	
	Indicator	Mean	Std. deviation	Mean	Std. deviation	F	Sig.
1	Physical well-being	49.38	6.00	53.10	4.25	6.312	0.015
2	Psychological well-being	55.86	7.22	54.85	6.11	0.283	0.598
3	Distress of treatment or illness	37.40	6.94	36.51	6.79	0.207	0.652
4	Fear	47.82	3.25	47.81	4.01	0.000	0.997
5	Social well-being	50.81	7.98	51.23	6.03	0.045	0.832
6	Spiritual well-being	52.40	6.79	49.21	5.96	3.075	0.086
7	Global quality of life score	49.54	2.99	49.42	2.29	0.022	0.883

The patients scored well when asked about their psychological well-being, with a total score of 55.31 ± 6.5. The mean score for difficulty in coping with the disease was 3.69 ± 1.58, and for change of self-concept was 3.69 ± 1.58 (Table 4).

**Table 4:** Quality of life subscale domain scores in relation to the psychological well-being of the patients

Sr. No.	Characteristic item	Mean	Standard deviation
1	Difficulty in coping as a result of disease	3.69	1.58
2	Difficulty in coping as a result of treatment	5.12	2.00
3	Quality of life	7.08	0.91
4	Happiness	7.08	0.91
5	In control of situations	6.94	0.90
6	Satisfying	4.96	0.89
7	Ability to concentrate or remember	6.02	0.85
8	Feeling of usefulness	6.02	0.85
9	Change in appearance	4.69	1.58
10	Change of own self-concept	3.69	1.58
Total	Standardized score	55.31	6.58

As presented in Table 5, overall patients were distressed with a standardized score of 36.91 ± 6.80. Psychological reactions to initial diagnosis as expected were mostly negative with a score of 2.5 ± 1.23; however, distress at chemotherapy was even more (1.56 ± 1.18); this was followed by anxiety at 3.08 ± 1.17. Distress regarding cancer surgery and completion of treatment was comparatively less with better scores of 4.51 ± 1.23 and 6.10 ± 1.12, respectively. Overall depression was less with a score of 5.02 ± 1.07.

**Table 5:** Quality of life subscale domain scores in relation to the distress of the patients

Sr. No.	Characteristic item	Mean	Std. deviation
1	Distress initial diagnosis	2.51	1.23
2	Chemotherapy	1.56	1.18
3	Cancer radiation	3.08	1.17
4	Cancer surgery	4.51	1.23
5	Completion of treatment	6.10	1.12
6	Anxiety	3.08	1.17
7	Depression	5.02	1.07
8	Standardized score	36.91	6.80

As given in Table 6, the patients had a moderate degree of fear with a score of 47.82 ± 3.65, with a maximum fear regarding recurrence and metastasis with scores of 2.45 ± 1.19 and 2.39 ± 1.22, respectively. They were less fearful regarding second cancer or future diagnostic tests. The patients were more or less confident regarding life getting back to normal with a score of 7.04 ± 0.73.

**Table 6:** Quality of life subscale domain scores in relation to fear in the patients

Sr. No.	Characteristic item	Mean	Std. deviation
1	Fear future diagnostic tests	5.92	1.17
2	Second cancer	6.11	1.22
3	Recurrence of cancer	2.45	1.19
4	Metastasis	2.39	1.22
5	Life getting back to normal	7.04	0.73
6	Standardized score	47.82	3.65

When the social concerns of breast cancer patients were assessed, it was found that they were most concerned regarding the financial burden on family, distress of illness on a family, and isolation due to illness. With scores of 3.76 ± 1.48, 3.80 ± 1.37, and 4.18 ± 0.78, they scored less when asked about the impact on sexuality (3.69 ± 1.58). Better scores were returned when the impact on employment, interference in a personal relationship, and impact on activities at home were enquired. It is demonstrated in Table 7.

**Table 7:** Quality of life subscale domain scores in relation to social well-being of the patients

Sr. No.	Characteristic Item (social concerns)	Mean	Std. deviation
1	Distress of illness on family	3.80	1.37
2	Support received	5.92	0.91
3	Interference in personal relationships	5.18	0.73
4	Impact on sexuality	3.69	1.58
5	Impact on employment	7.61	1.22
6	Impact on the activity at home	6.76	1.48
7	Isolation due to illness	4.18	0.73
8	Concern about daughter and female relatives	5.04	0.73
9	Financial burden	3.76	1.48
10	Standardized score	51.04	6.90

As presented in Table 8, overall spiritual well-being scores were better with a mean of 50.64 ± 6.48. Patients as expected could not harp on positive changes in life due to an illness score of 2.80 ± 0.68; however, they had the best scores regarding uncertainty about the future (6.45 ± 1.43). Participation in religious and spiritual activities revealed scores of 5.10 ± 1.42 and 5.41 ± 1.48.

**Table 8:** Quality of life subscale domain scores in relation to the spiritual well-being of the patients

Sr. No.	Characteristic item	Mean	Std. deviation
1	Participation in religious activities	5.10	1.42
2	Spiritual activities	5.41	1.48
3	Change in spiritual life due to diagnosis of cancer	5.10	1.42
4	Uncertainty about future	6.45	1.43
5	Positive changes in life due to illness	2.80	0.68
6	Impact on the sense of purposes of life	5.57	0.65
7	Hopeful	5.02	1.07
8	Standardized score	50.64	6.48

To study the impact of mastectomy on body image and sexuality, four items from the instrument were identified. It was found that scores for change in appearance, self-concept, and sexuality were very poor. Interference in interpersonal relationships was average. Values are mentioned in Table 9.

**Table 9:** Impact of mastectomy on body image and sexuality

Sr. No.	Characteristic Item	Mean	Std. deviation
1	Change in appearance	4.694	1.584
2	change in self-concept	3.694	1.584
3	Interference in personal relationship	5.184	0.727
4	Impact on sexuality	3.694	1.584

Few questions regarding treatment satisfaction and affordability were included in the study instrument, which is mentioned in Table 10. The majority of patients (71.43%) were satisfied with the treatment received. However, only 30% felt that it was affordable to them. Nearly half of the patients (44.9%), however, expressed confidence that somehow, they will be able to follow requisite follow-up guidelines and investigation. Of the patients, 36.73% were willing to undergo breast reconstruction; however, only 10.2% felt that they can afford it when informed about the cost of treatment.

**Table 10:** Treatment satisfaction and affordability

Sr. No.	Satisfaction/affordability	Yes		No	
		Number	Percentage	Number	Percentage
1	Satisfied with treatment	35	71.43	14	28.57
2	Was the treatment affordable?	15	30.61	34	69.39
3	Ability to follow up	22	44.9	27	55.10
4	Want to undergo breast reconstruction	18	36.73	31	63.27
5	Can afford reconstruction	5	10.2	34	69.39

## Discussion

The present study was conducted at a rural teaching institute in central Maharashtra, India. The aim was to assess the QOL in patients of mastectomy with chemotherapy. The QOL was evaluated using the QOL BC version [19]. It is an instrument developed by the City of Hope National Medical Center, which is free for use for research/clinical practice. Though the quality of assessment is a mandatory component of research in cancer and clinical trials, very limited information is available about the QOL in rural Indian patients.

The majority of patients presented after 50 years of age (55.10%), with a mean age of 51.35 ± 12.14 years. Pandey et al. [21] reported the mean age at presentation to be 47.6 ± 11 years. The majority of patients presented in stage III of the disease (61.22%), followed by the second stage (32.65%), with only 6.12% in stage 1 of the disease. This finding correlates well with those presented by Al Zahrani et al. [22], with 60% of their patients presenting in stage III of the disease.

The QOL was assessed on the QOL BC version. The QOL was divided into the physical domain (extent of the problem caused by fatigue, appetite changes, and aches and pain), psychological well-being, distressing aspects of illness of treatment and disease, fear of various factors, social concerns, and spiritual well-being. All the scores were standardized to get values out of 100. It was found that the total global score was nearly 50% (49.47 ± 2.6%). All individual domains had scores above 50%, except distress and fear. None of the Indian studies has used the present instrument for QOL analysis; however, global scores from other studies can be a pointer. The reliability and validity of the present instrument were tested by the City of Hope National Medical Center. Gangane et al. [23] reported that the mean score across all groups for QOL was 55.5, slightly higher than the present study. Ismaili et al. [24] have used other scores and they have found total health scores (mean: 57.2 ± 25.4), which are similar to the present study. In their study using the functional assessment of cancer therapy scores, Pandey et al. [21] found that QOL scores in breast cancer patients in all four domains of assessment were near median scores, which are near 50% of the maximum scores. It is reported that women with breast cancer achieve maximum psychological and physical recovery one year after the establishment of diagnosis [25,26]. All the patients in the present study were below one year after diagnosis, so the scores may be low.

The distress aspects covered questions like distress from radiotherapy, chemotherapy, surgery, distress because of prolonged treatment, anxiety, and depression. So the scores are lowest for this domain. It is reported that depression is quite common in breast cancer patients with nearly 42-48% of patients having mild to severe depression [26]. The present study was carried out in a rural population where the word cancer itself evokes negative responses. The excellent results of breast cancer are beyond the comprehension of patients. Coupled with the fact that advanced therapies are still not available and are costly may lead the treatment to become distressing.

The psychological well-being of breast cancer patients was assessed on a 10-item questionnaire. Women suffering from breast cancer tend to have a higher risk of psychological problems [27]. Psychological scores show the difficulty that patients had regarding insight into their disease. In a study by Al Zahrani et al. [22], raw mean scores of psychological well-being were reported to be 7.09 ± 0.30 (standardized score = 79). In the present study, the psychological score was found to be around 55 ± 6.58. Gangane et al. [23] reported a psychological well-being score of 58.2 on the World Health Organization Quality of Life (WHOQOL) scale, which corresponds to the present study. The participants had maximum difficulty in coping as a result of the disease (3.69 ± 1.58), and they felt that their life is less satisfying. Both of these point toward inner conflict rather than outward psychological pointers like the feeling of usefulness and happiness. It is a well-known fact that the initial diagnosis of cancer evokes a state of shock, fear, and disbelief, thus creating a psychological crisis [22].

Spiritual well-being is not a well-studied aspect of QOL. It was not mentioned in any of the QOL scales, except in the WHOQOL scale [23]. The QOL BC version assesses spiritual well-being. Seven items attempt to quantify spiritual well-being. In the present study, participants scored a normalized score of 50.64 ± 6.48 on this subscale. However, the response to the question regarding the extent to which your illness made positive changes to life attracted the lowest score of 2.80 ± 0.68. The average spiritual well-being score albeit with a different instrument was reported to be 65.91 ± 12.177 in western literature [28].

An analysis of QOL scores in different domains to demographic indicators failed to find a correlation with factors like age, disease status, religion, socioeconomic status, etc. This is contrary to what is reported in the literature [21,22]. Probably the effect of coronavirus disease 2019 (COVID-19) in the previous year might have led to a decrease in the QOL in these patients. All of the patients were less than one year from diagnosis of malignancy, which means, essentially, they have spent a whole year under the shadow of COVID-19.

Individual subscale analysis

The QOL BC version includes eight items for analysis of the physical domain of life quality. Fatigue, aches and pains, and vaginal dryness were the most dominant physical problems in breast cancer patients in the present study, with a mean of 3.10 ± 1.33, 3.69 ± 1.58, and 1.20 ± 0.87, respectively. Menstrual changes and sleep changes were the least bothersome (7.06 ± 2.54 and 7.78 ± 1.31), whereas appetite is among these two.

Fatigue and pain are included in the symptom scale of the European Organization for Research and Treatment of Cancer (EQRTC) scale. Mean fatigue and pain and insomnia have been reported to be 33.3 ± 30.1 and 34.3 ± 32.6, respectively, similar to the present findings [27]. Fatigue and pain are reported to be the main problems along with hair loss in patients with breast cancer. Wani et al. [29] reported that fatigue, insomnia, and pain, which are present at a mean of 33.34, 33.84, and 23.23 at the first visit, decrease subsequently by the second year after treatment.

Vaginal dryness is very infrequently studied in breast cancer, though it forms part of the menopause symptom scale. Morrow et al. [30] reported that it was present at a mean score of 1.4 ± 1.5 in a score range of zero to four, which is very high in young breast cancer patients and 1.6 ± 1.5 in elderly patients with breast cancer. Breast cancer treatment may precipitate menopause and this can be especially distressing to young breast cancer survivors.

The participants scored worst relating to questions related to distress. Though the depression score was not very bad (5.02 ± 1.07), every aspect of disease and treatment from diagnosis to chemotherapy, radiotherapy, and surgery was distressing for their scores for these were very low, suggesting a high degree of distress. The diagnosis and treatment of malignancies are tedious and demanding. Increased five-year survival rates and increased life expectancy have created a large number of breast cancer patients who are under treatment for a longer duration of time, putting extreme financial, emotional, and physical strain on the patients.

The part coping with fears of cancer patients deals with the fear of future diagnostic tests (5.9 ± 1.17), fear of second cancer (6.11 ± 1.22), recurrence (2.4 ± 1.19), and metastasis (2.39 ± 1.22), with an overall score of 47.82 ± 3.65. In other words, fear dominates cancer patients. Härtl et al. [31] report that fear of recurrence is the most important fear after cancer, even after long-term survival. Many of their patients, even though treated with mastectomy, reported fear of recurrence. It was present in 55.3% of patients undergoing mastectomy and 63.9% of patients undergoing breast conservation surgery. In nearly 40% of patients, the fear is so high that they need help in coming out of it.

Fear and distress together with few physical scores like vaginal dryness, fatigue, and sleep disturbances were the main factors in the present study that grossly affected the QOL of cancer patients in adverse ways.

The effect of breast cancer was assessed on body image and sexuality. Sexuality was considered from the participant's perspective. It was found that patients scored poorly on body image, sexuality, and self-concept scores. It is reported that mastectomy leads to poor body image. Western cancer survivors use breast reconstruction or at least external prostheses to improve body image. Härtl et al. [31] reported body image scores of 24.8 ± 26.1 in their study. Breast cancer management is associated with menopausal symptoms, vaginal dryness, and dyspareunia. These coupled with the loss of accessory sex organs in the form of the breast might induce a sense of incompleteness in females, explaining negative scores on self-concept and sexuality.

Affordability and satisfaction with a given treatment are difficult to quantify. Few questions were asked about the same, it was found that the majority of patients were satisfied with the treatment given to them; however, most of them felt that it was costly. Nearly 50% of patients expressed their ability to follow up, which is most important for long-term success. All the patients were explained in detail about the procedure, cost, and results of breast reconstruction; however, only one-third expressed their desire to undergo the same and further only one-tenth of them could afford the same. Apart from the advantages and disadvantages of breast reconstruction surgery, affordability remains the most important issue in the management of these patients in whom the majority of medical expenses are out-of-pocket expenses.

A few recommendations to be suggested are as follows: there is an urgent need to change the focus of cancer treatment from curative/palliative to improving QOL. Improved communication and counseling efforts to address the fear of various factors like future cancer and metastases in cancer survivors can decrease fear and improve QOL. Developing protocols to decrease avoidable distress during treatment like waiting for time, travel, etc. will also help in improving QOL.

The limitation is the sample size is small, so further research is necessary.

## Conclusions

The present study shows that the average QOL scores in rural Indian breast cancer patients undergoing mastectomy with chemotherapy are moderate. Global scores and other indicators show a moderate QOL. Distress because of disease and treatment-related factors along with various fears are the areas that bring down the QOL in these participants. Distress related to cancer diagnosis and treatment can be decreased by careful planning of treatment and adequate care during treatment implementation. Fear of various factors was one of the worse areas of QOL, which is more amenable to improvement by investing in patient counseling and giving confidence to the patient.

Fatigue, pains and aches, and vaginal dryness were the worst individual items where scores were very low. The patients scored poorly on body image and sexuality aspects. Contrary to the whole body of literature, we could not find any effect of sociodemographic factors on QOL. However, the study was conducted in the immediate post-COVID-19 era and the psychological, financial, and physical effects of COVID-19 were fresh in the mind of the patients. These effects might explain, to some extent, the moderate QOL across the board in these patients.

## Appendices

Questionnaire

Name:

Age:

Sex:

Religion:

Income:

Residence:

Registration No.:

Stage of the disease:

Procedure performed:

Type of chemotherapy: adjuvant/neoadjuvant:

Drugs used: CAF/AC/Paclitaxel or other:

Quality of Life Scale/Breast Cancer Patient

Directions: We are interested in knowing how your experience of having cancer affects your quality of life. Please answer all of the following questions based on your life at this time. Please circle the number from 0 to 10 that best describes your experiences:

• Physical changes

To what extent are the following a problem for you:

1. Fatigue:

No problem, 0, 1, 2, 3, 4, 5, 6, 7, 8, 9, 10, severe problem

2. Appetite changes:

No problem, 0, 1, 2, 3, 4, 5, 6, 7, 8, 9, 10, severe problem

3. Aches or pain:

No problem, 0, 1, 2, 3, 4, 5, 6, 7, 8, 9, 10, severe problem

4. Sleep changes:

No problem, 0, 1, 2, 3, 4, 5, 6, 7, 8, 9, 10, severe problem

5. Weight gain:

No problem, 0, 1, 2, 3, 4, 5, 6, 7, 8, 9, 10, severe problem

6. Vaginal dryness/menopausal symptoms:

No problem, 0, 1, 2, 3, 4, 5, 6, 7, 8, 9, 10, severe problem

7. Menstrual changes or fertility:

No problem, 0, 1, 2, 3, 4, 5, 6, 7, 8, 9, 10, severe problem

8. Rate your overall physical health:

Extremely poor, 0, 1, 2, 3, 4, 5, 6, 7, 8, 9, 10, excellent

• Psychological well-being items

9. How difficult is it for you to cope today as a result of your disease?

Not at all, 0, 1, 2, 3, 4, 5, 6, 7, 8, 9, 10, very difficult

10. How difficult is it for you to cope today as a result of your treatment?

Not at all, 0, 1, 2, 3, 4, 5, 6, 7, 8, 9, 10, very difficult

11. How good is your quality of life?

Not at all, 0, 1, 2, 3, 4, 5, 6, 7, 8, 9, 10, very difficult

12. How much happiness do you feel?

Not at all, 0, 1, 2, 3, 4, 5, 6, 7, 8, 9, 10, very difficult

13. Do you feel like you are in control of situations in your life?

Not at all, 0, 1, 2, 3, 4, 5, 6, 7, 8, 9, 10, very difficult

14. How satisfying is your life?

Not at all, 0, 1, 2, 3, 4, 5, 6, 7, 8, 9, 10, very difficult

15. How is your present ability to concentrate or to remember things?

Extremely poor, 0, 1, 2, 3, 4, 5, 6, 7, 8, 9, 10, excellent

16. How useful do you feel?

Not at all, 0, 1, 2, 3, 4, 5, 6, 7, 8, 9, 10, extremely

17. Has your illness or treatment caused changes in your appearance?

Not at all, 0, 1, 2, 3, 4, 5, 6, 7, 8, 9, 10, extremely

18. Has your illness or treatment caused changes in your self-concept (the way you see yourself)?

Not at all, 0, 1, 2, 3, 4, 5, 6, 7, 8, 9, 10, extremely

• ​​​​​​​How distressing were the following aspects of your illness and treatment?

19. Initial diagnosis:

Not at all, 0, 1, 2, 3, 4, 5, 6, 7, 8, 9, 10, extremely

20. Cancer chemotherapy:

Not at all, 0, 1, 2, 3, 4, 5, 6, 7, 8, 9, 10, extremely

21. Cancer radiation:

Not at all, 0, 1, 2, 3, 4, 5, 6, 7, 8, 9, 10, extremely

22. Cancer surgery:

Not at all, 0, 1, 2, 3, 4, 5, 6, 7, 8, 9, 10, extremely

23. Completion of treatment:

Not at all, 0, 1, 2, 3, 4, 5, 6, 7, 8, 9, 10, extremely

24. How much anxiety do you have?

Not at all, 0, 1, 2, 3, 4, 5, 6, 7, 8, 9, 10, extremely

25. How much depression do you have?

Not at all, 0, 1, 2, 3, 4, 5, 6, 7, 8, 9, 10, extremely

•​​​​​​​ ​​​​​​​To what extent are you fearful of:

26. Future diagnostic tests:

Not at all, 0, 1, 2, 3, 4, 5, 6, 7, 8, 9, 10, extremely

27. Second cancer:

Not at all, 0, 1, 2, 3, 4, 5, 6, 7, 8, 9, 10, extremely

28. Recurrence of cancer:

Not at all, 0, 1, 2, 3, 4, 5, 6, 7, 8, 9, 10, extremely

29. Spreading (metastasis) of your cancer:

Not at all, 0, 1, 2, 3, 4, 5, 6, 7, 8, 9, 10, extremely

30. To what degree do you feel your life is back to normal?

Not at all, 0, 1, 2, 3, 4, 5, 6, 7, 8, 9, 10, extremely

•​​​​​​​ ​​​​​​​Social concerns

31. How distressing has your illness been for your family?

Not at all, 0, 1, 2, 3, 4, 5, 6, 7, 8, 9, 10, extremely

32. Is the amount of support you receive from others sufficient to meet your needs?

Not at all, 0, 1, 2, 3, 4, 5, 6, 7, 8, 9, 10, extremely

33. Is your continuing health care interfering with your personal relationships?

Not at all, 0, 1, 2, 3, 4, 5, 6, 7, 8, 9, 10, extremely

34. Is your sexuality impacted by your illness?

Not at all, 0, 1, 2, 3, 4, 5, 6, 7, 8, 9, 10, extremely

35. To what degree has your illness and treatment interfered with your employment?

Not at all, 0, 1, 2, 3, 4, 5, 6, 7, 8, 9, 10, extremely

36. To what degree has your illness and treatment interfered with your activities at home?

Not at all, 0, 1, 2, 3, 4, 5, 6, 7, 8, 9, 10, extremely

37. How much isolation do you feel is caused by your illness?

Not at all, 0, 1, 2, 3, 4, 5, 6, 7, 8, 9, 10, extremely

38. How much concern do you have for your daughter(s) or other close female relatives regarding breast cancer?

Not at all, 0, 1, 2, 3, 4, 5, 6, 7, 8, 9, 10, extremely

39. How much financial burden have you incurred as a result of your illness and treatment?

Not at all, 0, 1, 2, 3, 4, 5, 6, 7, 8, 9, 10, extremely

•​​​​​​​ Spiritual well-being

40. How important to you is your participation in religious activities such as praying and going to church or temple?

Not at all, 0, 1, 2, 3, 4, 5, 6, 7, 8, 9, 10, extremely

41. How important to you are other spiritual activities such as meditation or praying?

Not at all, 0, 1, 2, 3, 4, 5, 6, 7, 8, 9, 10, extremely

42. How much has your spiritual life changed as a result of your cancer diagnosis?

Not at all, 0, 1, 2, 3, 4, 5, 6, 7, 8, 9, 10, extremely

43. How much uncertainty do you feel about your future?

Not at all, 0, 1, 2, 3, 4, 5, 6, 7, 8, 9, 10, extremely

44. To what extent has your illness made positive changes in your life?

Not at all, 0, 1, 2, 3, 4, 5, 6, 7, 8, 9, 10, extremely

45. Do you sense a purpose/mission for your life or a reason for being alive?

Not at all, 0, 1, 2, 3, 4, 5, 6, 7, 8, 9, 10, extremely

46. How hopeful do you feel?

Not at all, 0, 1, 2, 3, 4, 5, 6, 7, 8, 9, 10, extremely

•​​​​​​​ Assessment of treatment offered at center and view regarding breast reconstruction surgery:

Are you satisfied with your treatment in the form of mastectomy?

Is treatment affordable to you or are you a government scheme patient?

Will you like to undergo another surgery for breast reconstruction?

Can you able to undergo regular follow-up for local recurrence?

Is the cost of reconstruction surgery affordable to you?
